# Optimizing Silicon Oxide Embedded Silicon Nanocrystal Inter-particle Distances

**DOI:** 10.1186/s11671-016-1567-6

**Published:** 2016-08-04

**Authors:** Martijn van Sebille, Jort Allebrandi, Jim Quik, René A.C. M. M. van Swaaij, Frans D. Tichelaar, Miro Zeman

**Affiliations:** 1 Photovoltaic Materials and Devices, Delft University of Technology, Mekelweg 4, Delft, 2628 CD The Netherlands; 2 Kavli Institute of Nanoscience, Quantum Nanoscience, Delft University of Technology, Lorentzweg 1, Delft, 2628 CJ The Netherlands

**Keywords:** Silicon nanocrystal, Silicon oxide, Inter-particle distance, Stoichiometry, Spacing

## Abstract

We demonstrate an analytical method to optimize the stoichiometry and thickness of multilayer silicon oxide films in order to achieve the highest density of non-touching and closely spaced silicon nanocrystals after annealing. The probability of a nanocrystal nearest-neighbor distance within a limited range is calculated using the stoichiometry of the as-deposited film and the crystallinity of the annealed film as input parameters. Multiplying this probability with the nanocrystal density results in the density of non-touching and closely spaced silicon nanocrystals. This method can be used to estimate the best as-deposited stoichiometry in order to achieve optimal nanocrystal density and spacing after a subsequent annealing step.

## Background

Silicon nanocrystals embedded in a high band gap silicon alloy are interesting candidates for top cells of multi-junction solar cells of which the band gap can be tuned by the nanocrystal size [1]. The ability to tune the material’s band gap allows us to minimize thermalization losses and thereby increase the solar cell efficiency. The mean nanocrystal size and size distribution are crucial parameters in determining the optical properties of the material [2, 3] and electronic transport properties in photovoltaic devices [4].

Embedded silicon nanocrystals can be made by annealing silicon-rich silicon alloy films, and this is typically performed using a tube furnace or rapid thermal annealing furnace [5]. Upon annealing at temperatures between 600 and 900 °C, phase separation of the excess silicon occurs, creating amorphous silicon nanoparticles surrounded by an amorphous silicon oxide matrix. Annealing at temperatures in excess of 900 °C leads to crystallization of these amorphous nanoparticles [6].

Using films containing alternating layers of stoichiometric and silicon-rich silicon alloys allows for the control over the nanocrystal size, limited by the silicon-rich layer thickness [7, 8]. Various charge transport mechanisms for embedded silicon nanocrystal have been suggested, including direct tunneling [9], trap-assisted tunneling [10], and hopping [11]. No clear consensus exists concerning the exact mechanisms, especially concerning the role of defects in the matrix and at the nanocrystal interface [10–14]. Nonetheless, the total charge transport is expected to be highly dependent on the nanocrystal spacing and the choice of dielectric material [15].

For SiO _2_ films, inter-particle spacing up to 2 nm is acceptable, which provides a minimum mobility of 10^−1^ cm^2^ V^−1^s, as calculated by Green et al. [1].

**Fig. 1:**
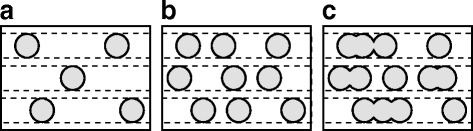
Nanocrystal spacing and clustering. Nanocrystals formed in silicon-rich layers with relatively low (**a**), medium (**b**), and high (**c**) excess silicon, separated by stoichiometric buffer layers

The nanocrystal density in the silicon-rich layers can be controlled by tuning the composition of these layers during deposition. A low silicon content leads to relatively few isolated nanocrystals, and increasing the excess silicon content will eventually lead to clustering of nanocrystals, shown schematically in Fig. 1.

When the nanocrystal density is too low, the probability of nearest-neighbor nanocrystal within 2 nm is too low. In contrast, when the excess silicon content is too high, nanocrystals are so closely spaced that they start clustering, which reduces the quantum confinement in these crystals. This means there is an optimal composition to achieve a limited nanocrystal spacing, while limiting clustering. In this letter, we demonstrate an analytical method to optimize the composition and thickness of multilayer silicon oxide films in order to achieve the highest density of non-touching and closely spaced silicon nanocrystals after annealing.

## Methods

We deposited approximately 100-nm-thick a-SiO _*x*_:H films on Spectrosil 2000 quartz substrates in a radio frequency plasma-enhanced chemical vapor deposition (PECVD) reactor, operating at 13.56 MHz. The following deposition parameters were used: a power density of 2.1×10^−2^ W cm ^−2^, a deposition pressure of 1.4 mbar, and a substrate temperature of 95 °C. The film composition was varied by changing the SiH_4_ over CO_2_ flow ratio from 0.07 to 0.37. A H_2_ flow rate of 200 sccm was used for all depositions. n- and p-type films were fabricated by including PH_3_ and B_2_H_6_ flows, respectively. The dopant over SiH_4_ flow rate ratio was 2.0×10^−3^. The atomic compositions of the silicon-rich and buffer layers were determined using x-ray photoelectron spectroscopy measurements, using a Thermo Scientific K-Alpha setup. The film surface was etched with an ion gun prior to measurements to remove surface contamination. Annealing was carried out using a Tempress horizontal tube stack or a Solaris 100 RTA furnace for 1 h and 3 min, respectively. All samples were annealed at 1000 °C, at atmospheric pressure and in pure nitrogen gas. The composition of the buffer layer used in these experiments is SiO _1.3_. Measurements show that this stoichiometry is sufficiently high to prevent crystallization for the annealing conditions used (not shown here). Raman spectra were measured to determine the crystallinity, using a Renishaw inVia setup in backscattering geometry, with a 25-mW Ar laser as excitation source with a wavelength of 514 nm and focused on a spot of approximately 1 *μ*m. The crystallinity *X*_C_ is the ratio of the Si–Si bonds in crystalline phase over the Si–Si bonds in amorphous and crystalline phase [16] and is calculated as follows:

1 $$ X_{\text{C}} = \frac{I_{\text{TO,c-Si}}}{\sigma I_{\text{TO,a-Si}} + I_{\text{TO,c-Si}}},  $$

where *I*_TO,c-Si_ and *I*_TO,a-Si_ are the integrated TO phonon modes of crystalline and amorphous silicon, respectively. *σ* is a factor to correct for the difference in scattering cross section between these modes and is set to 0.8 [16].

Imaging the silicon crystals in the amorphous silicon layer was done using a FEI Tecnai F20ST/STEM transmission electron microscope (TEM) operated at 200 kV. Thin samples for TEM were prepared in cross section following a standard procedure after gluing the two samples together face to face: a 500- *μ*m-thick lamella was cut out using a diamond saw, subsequently thinned to approximately 15- *μ*m thickness by mechanical polishing, glued on a copper support ring, and argon ion-milled to electron transparency. The silicon nanocrystals were marked using the freehand selection tool in ImageJ [17]. The surface area was then determined, and an effective diameter was recorded.

## Results and Discussion

**Fig. 2:**
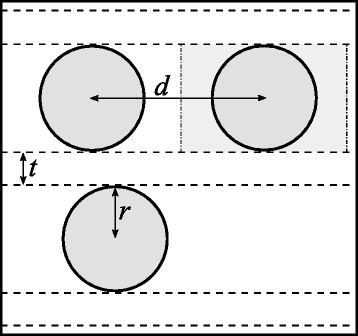
Nanocrystals in a multilayer structure shown schematically. Nanocrystals in a multilayer structure shown schematically, including the nanocrystal radius *r*, buffer layer thickness *t*, and inter-particle distance *d*. The *enclosing box* around a nanocrystal is shown for the right-hand nanocrystal

Figure 2 illustrates nanocrystals with radius *r* in a multilayer structure, including their parameters needed to determine the inter-particle distance *d*.

**Fig. 3:**
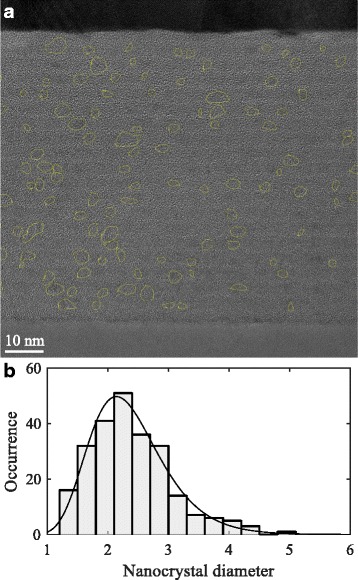
TEM image of a multilayer sample and its nanocrystal diameter histogram. **a** Cross-sectional high-resolution TEM image of an annealed multilayer sample with silicon-rich and buffer layer thicknesses of 3 and 1 nm, respectively. **b** The histogram of the sample’s nanocrystal diameters. Approximately 250 nanocrystals were measured. The histogram is fitted with a log-normal probability density function with *μ*= 0.83 nm and *σ*= 0.27 nm

We assume that the mean nanocrystal diameter equals the silicon-rich layer thickness. In order to validate this assumption, a multilayer sample with silicon-rich and buffer layer thicknesses of 3 and 1 nm, respectively, has been measured with high-resolution TEM, shown in Fig. 3a.

**Fig. 4:**
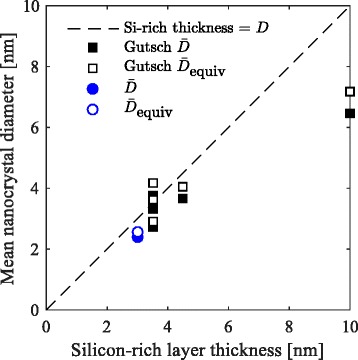
Nanocrystal diameter in a multilayer sample as a function of the silicon-rich layer thickness. The mean nanocrystal diameter $\bar {D}$ (*solid symbols*) and the mean equivalent diameter $\bar {D}_{\text {equiv}}$ (*open symbols*) for samples with varying silicon-rich layer thicknesses. The *black data points* are obtained from Gutsch et al. [18]. The *dashed line* represents the equality between the nanocrystal diameter and the silicon-rich layer thickness

The histogram of the obtained nanocrystal diameters is shown in Fig. 3b. The mean nanocrystal diameter obtained from TEM is 2.4 nm. Figure 4 shows the mean nanocrystal diameter as a function of its silicon-rich layer thickness of this sample, as well as data obtained by Gutsch et al. [18].

For thin silicon-rich layer thicknesses, the deviation between the mean nanocrystal diameter and the sample’s silicon-rich layer thickness is reasonable. For thicker layers, the deviation increases. However, we should note that instead of being interested in nanocrystal diameters, we are interested in the volume these nanocrystals occupy, since that allows us to predict the nanocrystal density and their inter-particle distance for varying stoichiometries and crystallinities. The size distribution of such multilayer samples is log-normally shaped, as was observed by Gutsch et al. [18] and can be seen from Fig. 3b. Since the volume of the nanoparticles depends on the third power of their radius, the larger nanocrystals have a greater contribution to the mean volume $\bar {V}$. This is given by

2 $$ \bar{V} = \frac{\sum\frac{4}{3}\pi r^{3}}{n_{\text{NC}}},   $$

where *r* and *n*_NC_ are the nanocrystal radius and the number of nanocrystals obtained from TEM, respectively. The equivalent diameter of the mean nanocrystal $\bar {D}_{\text {equiv}}$ can be expressed by

3 $$ \bar{D}_{\text{equiv}} = 2\sqrt[3]{\frac{\bar{V}}{\frac{4}{3}\pi}}.   $$

Combining Eqs. (2) and (3) results in

4 $$ \bar{D}_{\text{equiv}} = 2 \sqrt[3]{ \left\langle r^{3} \right\rangle},   $$

where 〈*r*^3^〉 represents the mean value of *r*^3^. The equivalent diameter of the sample shown in Fig. 3 is 2.6 nm and is shown in Fig. 4 along with the equivalent diameters of the data obtained by Gutsch et al. [18]. Because of the asymmetrical, log-normally shaped nanocrystal size distributions, all equivalent diameters are greater than their corresponding mean diameters. In general, the equivalent diameters are very close to the assumed equality between the nanocrystal diameter and the silicon-rich layer thickness. This result implies that our assumption is reasonable, at least up to silicon-rich layer thicknesses up to 4.5 nm. Fortunately, this range is most interesting for photovoltaic purposes because of their increased confinement.

Note that we do not include a core/shell structure in this approach. An amorphous sub-oxide shell is likely to form around silicon nanocrystals [7, 19]. Iacona et al. measured a shell to be approximately 1 nm thick [20]. This thickness corresponds with theoretical calculations and experimental measurements by Daldosso et al. [21]. However, Queeney et al. determined the shell thickness to be less than 6 Å [22]. Zimina et al. measured shell thicknesses of 2 to 5 Å and suggested a dependence on nanocrystal size [23]. These disagreeing results complicate the incorporation of a core/shell structure in our method. Since the aim of this publication is to provide a simple method to predict the inter-nanocrystal distance, we do not include the core/shell structure.

In order to optimize the density of non-touching and closely spaced silicon nanocrystals, the nanocrystal density in the silicon-rich layers should be determined first. This depends on the excess silicon in these layers and can be calculated from its composition as follows:

5 $$ \text{SiO}_{x} \rightarrow {\frac{{x}}{2}} \text{SiO}_{2} + \left(1-{\frac{x}{2}}\right)\text{Si}.   $$

The excess silicon can be in amorphous or crystalline phase, so the total atomic density in the layer *ρ*_layer_ is given by

6 $$ \rho_{\text{layer}} = \Gamma_{\text{c-Si}} \rho_{\text{c-Si}} + \Gamma_{\text{a-Si}} \rho_{\text{a-Si}} + \Gamma_{\text{SiO}_{2}} \rho_{\text{SiO}_{2}},   $$

where *ρ*_c-Si_ and *ρ*_a-Si_ are the atomic densities of c-Si and a-Si, respectively; $\rho _{\text {SiO}_{2}}\phantom {\dot {i}\!}$ is the molecular density of SiO _2_; and *Γ*_c-Si_, *Γ*_a-Si_, and $\Gamma _{\text {SiO}_{2}}\phantom {\dot {i}\!}$ are their respective atomic and molecular percentages. Using the definition of crystallinity and Eq. (5), the atomic percentages of c-Si, a-Si, and SiO _2_ can be written as

7 $$\begin{array}{@{}rcl@{}} \Gamma_{\text{c-Si}} & = & X_{\text{C}} \left(1- \frac{x}{2}\right) \cdot 100 \% \end{array} $$

8 $$\begin{array}{@{}rcl@{}} \Gamma_{\text{a-Si}} & = & \left(1-X_{\text{C}}\right) \left(1-\frac{x}{2}\right) \cdot 100 \% \end{array} $$

9 $$\begin{array}{@{}rcl@{}} \Gamma_{\text{SiO}_{2}} & = & \frac{x}{2} \cdot 100 \% \end{array} $$

The atomic density of c-Si in the layer can then be determined by

10 $$ \rho_{\text{c-Si,layer}} = \Gamma_{\text{c-Si}} \rho_{\text{layer}}.  $$

The number of atoms in a nanocrystal is $N_{\text {NC}} = \frac {4}{3}\pi r^{3} \rho _{\text {c-Si}}$, which can be used to calculate the 2D nanocrystal density *n*_2D_ in a silicon-rich layer with thickness 2*r*

11 $$ n_{2\text{D}} = \frac{\rho_{\text{c-Si,layer}}}{N_{\text{NC}}}2r.  $$

**Fig. 5:**
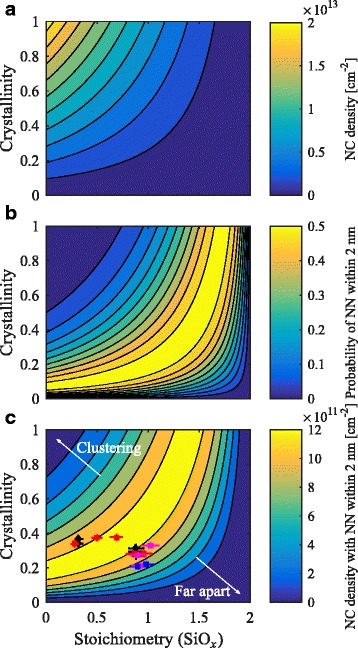
Nanocrystal density, NN probability, and NN density. The 2D nanocrystal (NC) density (**a**), the probability of finding a nearest neighbor (NN) within 2 nm (**b**), and the density of nanocrystals with a NN within 2 nm (**c**) as a function of the silicon-rich layer composition and crystallinity for a sample with silicon-rich and buffer layer thicknesses of 3 and 1 nm, respectively. The *black diamonds* represent tube furnace annealed intrinsic samples. The *red*, *magenta*, and *blue squares* show intrinsic, p-type and n-type samples annealed using RTA

Figure 5a shows the 2D nanocrystal density for a sample with silicon-rich and buffer layer thicknesses of 3 and 1 nm, respectively, using *ρ*_c-Si_, *ρ*_a-Si_, and $\rho _{\text {SiO}_{2}}\phantom {\dot {i}\!}$ 5.0×10^28^, 5.0×10^28^, and 2.2×10^28^ m^−3^, respectively [24–27].

The 2D nanocrystal density is highest for silicon-rich layers with high crystallinity and low stoichiometry. However, in that case, the nanocrystal density can be so high that crystals cluster together as illustrated in Fig. 1c. To find the fraction of nanocrystals that are properly spaced, we use the probability density function $\mathcal {F}$ of finding a nearest neighbor at distance *d* for a nanocrystal in a multilayer sample. For randomly distributed point particles, this is given by [28]

12 $$\begin{array}{@{}rcl@{}}{} \mathcal{F}\left(d\right) = \left(4j+2 \right) n_{2\text{D}} \pi d \exp\left[ -\left(2j+1\right) n_{2\text{D}} \pi d^{2} \right]\times  \\* \exp\left[ \frac{n_{2\text{D}} \pi t^{2} j \left(j+1\right) \left(2j+1\right)}{3}\right],  \end{array} $$

where *t* is the buffer layer thickness and *j*=⌊*r*/*t*⌋. Note that this probability density function describes center-to-center distances. Integrating this equation from 2*r* to 2*r*+2 nm provides the edge-to-edge probability of finding a nearest neighbor within 2 nm for a multilayer structure with silicon-rich layer thickness of 3 nm. This is shown in Fig. 5b and confirms that the optimal probability of finding the nearest neighbor between 0 to 2 nm is very low in the range where the 2D nanocrystal density is highest, caused by clustering of the nanocrystals. We can find an optimum stoichiometry for a given crystallinity using the result shown in Fig. 5b, but aside from proper spacing of nanocrystals, we are also interested in a high nanocrystal density. Integrating Eq. (12) and multiplying with the 2D nanocrystal density provides the non-touching nanocrystal density with a nearest neighbor within *d*

13 $$ n_{\text{NN}}\left(d\right) = n_{2\text{D}} {\int\nolimits}_{2r}^{2r+d} \mathcal{F}\left(d\right) \text{d}d.   $$

The nanocrystal density with a nearest neighbor within 2 nm for a sample with silicon-rich layer thickness of 3 nm is shown in Fig. 5c. The highest non-touching nanocrystal density for this structure can be achieved by tuning the silicon-rich layer’s composition from pure Si to approximately SiO _1.5_, with crystallinity values of 0.15 to 1, respectively. A too high crystallinity for layers with relatively low stoichiometry will lead to clustering, while too low crystallinity in layers with high stoichiometry will result in separated but too isolated nanocrystals.

We deposited multilayer films with silicon-rich layer and buffer layer thicknesses of 3 and 1 nm, respectively, and varied the silicon-rich layer’s compositions and doping. The films were annealed in a tube furnace or rapid thermal annealing furnace. The crystallinity of these films after annealing are shown in Fig. 5.^1^ The crystallinity in our films does not exceed 0.4. We expect that this is caused by incomplete phase separation prior to crystallization, as observed before for silicon oxide films deposited using PECVD [29]. This means that only part of all excess silicon (see Eq. (5)) clusters into silicon nanoparticles, which can subsequently crystallize. The rest of the excess silicon remains in the surrounding matrix, which will not be SiO_2_, but has a lower stoichiometry. This in turn will lower its energy barrier, increasing the tunneling probability and possibly enlarge the inter-particle distance at which the mobility remains sufficiently high [30]. However, for simplicity, we will keep an inter-particle distance range from 0 to 2 nm. Assuming a maximum achievable crystallinity of 0.4 for PECVD films, an optimal stoichiometry to achieve the highest density of non-touching, closely spaced nanocrystals can be found. This optimal stoichiometry is SiO _0.84_. In contrast, films deposited using magnetron sputtering are reported to lead to complete phase separation [29]. Assuming all silicon clusters crystallize upon annealing, this will lead to a crystallinity equal to unity. In reality, the sub-oxide shell around the nanocrystal core will limit complete crystallization [31], but for simplicity, we assume a crystallinity equal to unity. In that case, the optimal stoichiometry of the silicon-rich layers is approximately SiO _1.4_ for this structure. Note that in both cases, the 2D nanocrystal density with nearest neighbor within 2 nm is 1.3×10^12^ cm^−2^. This value corresponds well with results obtained experimentally by Laube et al. for single 4.5-nm-thick layers [32] and is slightly lower than experimental results obtained by Gutsch et al. for single 3.5-nm-thick layers [18]. However, we should note that these reported values are the total nanocrystal density, while we estimated the isolated nanocrystal density with nearest neighbor within 2 nm. This excludes clustered nanocrystals and too isolated nanocrystals, which inevitably leaves out a portion of the total nanocrystal density. Furthermore, the thickness of the silicon-rich layer affects the estimated nanocrystal density, with lower values for thicker layers.

**Fig. 6:**
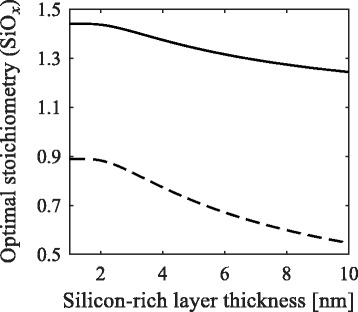
Optimal stoichiometry. The optimal stoichiometry for films with varying silicon-rich layer thicknesses for a sample with crystallinity of 1 (*solid line*) and 0.4 (*dashed line*). The buffer layer thickness is kept constant at 1 nm

The optimal stoichiometry decreases for increasing silicon-rich layer thicknesses, as shown in Fig. 6.

**Fig. 7:**
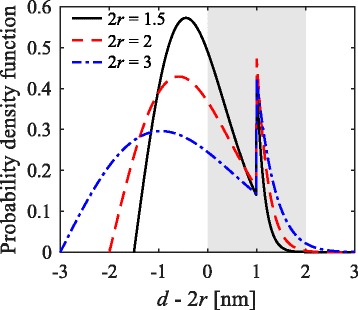
Nearest-neighbor distance probability density functions. The nearest-neighbor distance probability density functions for samples with silicon-rich layer thicknesses of 1.5, 2, and 3 nm and buffer layer thickness of 1 nm, calculated using their respective optimal compositions. The *gray area* depicts the range of desired nearest-neighbor distances. Shorter distances imply clustering, and greater distances lead to an insufficient tunneling probability

Note that the model’s accuracy decreases for greater silicon-rich layers thicknesses, as shown in Fig. 4. Nonetheless, from a theoretical perspective, the observed trend for thick silicon-rich layers is still interesting. This is caused by the differences in volume between a nanocrystal and its enclosing box (see Fig. 2). The volume of a nanocrystal is $\frac {4}{3}\pi r^{3}$, and its enclosing box is approximately 2*r*(2*r*+*d*)^2^. The volume ratio of the enclosing box over the nanocrystal decreases with increasing silicon-rich layer thickness, explaining the trend observed in Fig. 6 for relatively large silicon-rich layer thicknesses. However, for very small silicon-rich layer thicknesses, the optimal composition does not vary. To explain this, we look closer into their probability density functions, shown in Fig. 7.

The peak at 1 nm is caused by the availability of nearest neighbors in the neighboring silicon-rich layers. The probability density function broadens for larger silicon-rich layer thicknesses because its standard deviation is related to the 2D nanocrystal density by $\sigma _{2\text {D}} \propto 1/\sqrt {n_{2\text {D}}}$ [28]. Since *n*_2D_ decreases for greater silicon-rich layer thicknesses, the probability density functions broaden. The optimal composition depends on the probability of finding a nearest neighbor within a limited range (2 nm for SiO _*x*_). For silicon-rich layer thicknesses up to approximately 2 nm, the probability of finding a nearest neighbor beyond 2 nm is negligible, as can be observed in Fig. 7. For these thicknesses, there is no reason to increase the nanocrystal density, since more closely packed nanocrystals will not increase the probability of finding a nearest neighbor within 2 nm. On the contrary, an increase in nanocrystal density will increase the probability of clustering.

## Conclusions

We demonstrated an analytical method to optimize the composition of a silicon-rich layer for different crystallinities and thicknesses in order to achieve the highest density of non-touching and closely spaced silicon nanocrystals after annealing. The optimal stoichiometry depends on the crystallinity decreases for increasing silicon-rich layer thicknesses. However, for very small silicon-rich layer thicknesses, the optimal composition does not vary. This method can be used to find the best as-deposited composition in order to achieve optimal nanocrystal density and spacing after a subsequent annealing step.

## Endnote

^1^ Note that the buffer layer composition is not stoichiometric and therefore will contain some excess silicon as well. This excess silicon will increase the amorphous Si–Si bond density, resulting in an underestimation of the crystallinity. However, since the buffer layer thickness is only 1 nm, compared to 3 nm for the silicon-rich layer, we expect this effect to be limited and assume it can be neglected.
